# Clinical phenotypes and survival of pre-capillary pulmonary hypertension in systemic sclerosis

**DOI:** 10.1371/journal.pone.0197112

**Published:** 2018-05-15

**Authors:** David Launay, David Montani, Paul M. Hassoun, Vincent Cottin, Jérôme Le Pavec, Pierre Clerson, Olivier Sitbon, Xavier Jaïs, Laurent Savale, Jason Weatherald, Vincent Sobanski, Stephen C. Mathai, Majid Shafiq, Jean-François Cordier, Eric Hachulla, Gérald Simonneau, Marc Humbert

**Affiliations:** 1 Univ. Lille, U995, Lille Inflammation Research International Center (LIRIC), Lille, France; 2 Inserm, U995, Lille, France; 3 CHU Lille, département de médecine interne et immunologie clinique, Lille, France; 4 Centre national de référence maladies systémiques et auto-immunes rares (sclérodermie systémique), Lille, France; 5 Université Paris-Sud, Faculté de Médecine, Université Paris-Saclay, Le Kremlin-Bicêtre, France; 6 Service de Pneumologie, Hôpital Bicêtre, Assistance Publique Hôpitaux de Paris, Le Kremlin-Bicêtre, France; 7 INSERM UMR S 999, Hôpital Marie Lannelongue, Le Plessis Robinson, France; 8 Division of Pulmonary and Critical Care, Department of Medicine, Johns Hopkins University, Baltimore, MD, United States of America; 9 Centre national de référence des maladies pulmonaires rares, hôpital Louis Pradel, Hospices Civils de Lyon, Université Claude Bernard Lyon 1, Lyon, France; 10 Service de Chirurgie Thoracique, Vasculaire et Transplantation Cardio-pulmonaire, Hôpital Marie Lannelongue, Le Plessis Robinson, France; 11 Soladis Clinical Study, Roubaix, France; 12 Division of Respirology, Department of Medicine, University of Calgary, Calgary, Alberta, Canada; Vanderbilt University Medical Center, UNITED STATES

## Abstract

Pre-capillary pulmonary hypertension (PH) in systemic sclerosis (SSc) is a heterogeneous condition with an overall bad prognosis. The objective of this study was to identify and characterize homogeneous phenotypes by a cluster analysis in SSc patients with PH. Patients were identified from two prospective cohorts from the US and France. Clinical, pulmonary function, high-resolution chest tomography, hemodynamic and survival data were extracted. We performed cluster analysis using the k-means method and compared survival between clusters using Cox regression analysis. Cluster analysis of 200 patients identified four homogenous phenotypes. Cluster C1 included patients with mild to moderate risk pulmonary arterial hypertension (PAH) with limited or no interstitial lung disease (ILD) and low DLCO with a 3-year survival of 81.5% (95% CI: 71.4–88.2). C2 had pre-capillary PH due to extensive ILD and worse 3-year survival compared to C1 (adjusted hazard ratio [HR] 3.14; 95% CI 1.66–5.94; p = 0.0004). C3 had severe PAH and a trend towards worse survival (HR 2.53; 95% CI 0.99–6.49; p = 0.052). Cluster C4 and C1 were similar with no difference in survival (HR 0.65; 95% CI 0.19–2.27, p = 0.507) but with a higher DLCO in C4. PH in SSc can be characterized into distinct clusters that differ in prognosis.

## Introduction

Pulmonary hypertension (PH) is a severe complication of systemic sclerosis (or scleroderma) (SSc) affecting more than 10% of patients during their lifespan [1]. PH is defined by right-heart catheterization showing an elevated mean pulmonary artery pressure (PAP) ≥ 25 mmHg [2]. In patients with SSc, PH may result from several causes and mechanisms[2, 3]. Pre-capillary PH (pulmonary artery wedge pressure ≤ 15 mmHg) can also occur, most often in the setting of group 1 (PAH), but also of group 1’ (pulmonary veno-occlusive disease and/or pulmonary capillary hemangiomatosis), group 3 (PH due to chronic lung diseases), or group 4 (chronic thrombo-embolic PH) [2, 4]. Importantly, clinical management varies markedly by the cause of PH, with different treatment indicated for each subgroup of patients.

Despite the advent of targeted therapies approved for pulmonary arterial hypertension (PAH), PH is still a leading cause of mortality in SSc [5]. The prognosis of SSc-associated PAH remains poor and survival is worse than in idiopathic PAH [6, 7]. Among the possible explanations for such poor outcomes, the high degree of heterogeneity of SSc must be highlighted. Indeed, SSc is a multisystem disease characterized by an extensive vasculopathy, inflammation and variable fibrosis affecting not only the entire pulmonary vasculature but also the lung parenchyma (with up to 50% of patients presenting with interstitial lung disease (ILD)[8].

In clinical practice, it may be challenging to classify patients with SSc in a discrete PH group because of complex and frequently overlapping clinical features. Indeed, in the setting of pre-capillary PH, SSc patients may present with no, limited, or extensive ILD and a wide range of hemodynamic severity [9–11]. However, classification of these patients remains meaningful, as medical therapies are dependent on it. For example, drugs approved for PAH[12] may be deleterious in patients with pulmonary veno-occlusive disease or PH due to chronic lung diseases. The latter patients should be offered long-term oxygen therapy and/or referred for lung transplantation [2, 13].

Therefore, pre-capillary PH in SSc is highly heterogeneous both in terms of pathogenic mechanisms, including the presence and extension of ILD, and due to the heterogeneity of the underlying disease. One of the most useful methods for grouping patients into homogeneous subsets is the cluster analysis, the purpose of which is to identify phenotypic groups within a heterogeneous medical condition. Cluster analysis can identify relevant clinical features to be used in personalized management strategies as well as to understand the relationships between clinical features and outcome variables [14]. The primary objective of our present study was to identify and characterize homogeneous phenotypes by a cluster analysis in two independent, prospectively constituted US and French cohorts of SSc patients with pre-capillary PH. The secondary objective was to assess survival in the different clusters.

## Methods

### Inclusion criteria

Two cohorts of patients recruited between 1999 to 2011 from two independent French and US populations were analyzed. The French cohort was recruited in the Registry of the national Pulmonary Hypertension Network, which enrolls consecutive patients aged ≥18 years with PH. This was a retrospective study which complied with the Declaration of Helsinki. All data were anonymised and compiled according to the requirements of the Commission Nationale Informatique et Liberté, the committee dedicated to privacy, information technology and civil rights in France. The committee approved the methods used to collect and analyze registry data on May 24, 2003 (approval number 842063). Data from the three largest centers recruiting patients with SSc were used in this analysis (Le Kremlin-Bicêtre, Lille, and Lyon University Hospitals). Patients from the US cohort were identified from a group of prospectively enrolled patients into the Hopkins Pulmonary Hypertension Registry in the Division of Pulmonary and Critical Care Medicine at Johns Hopkins Hospital (Baltimore, USA). The Johns Hopkins Medicine Institutional Review Board (JHMIRB) approved the study (NA_00027124) and informed consent was obtained from all patients.

**Fig 1:**
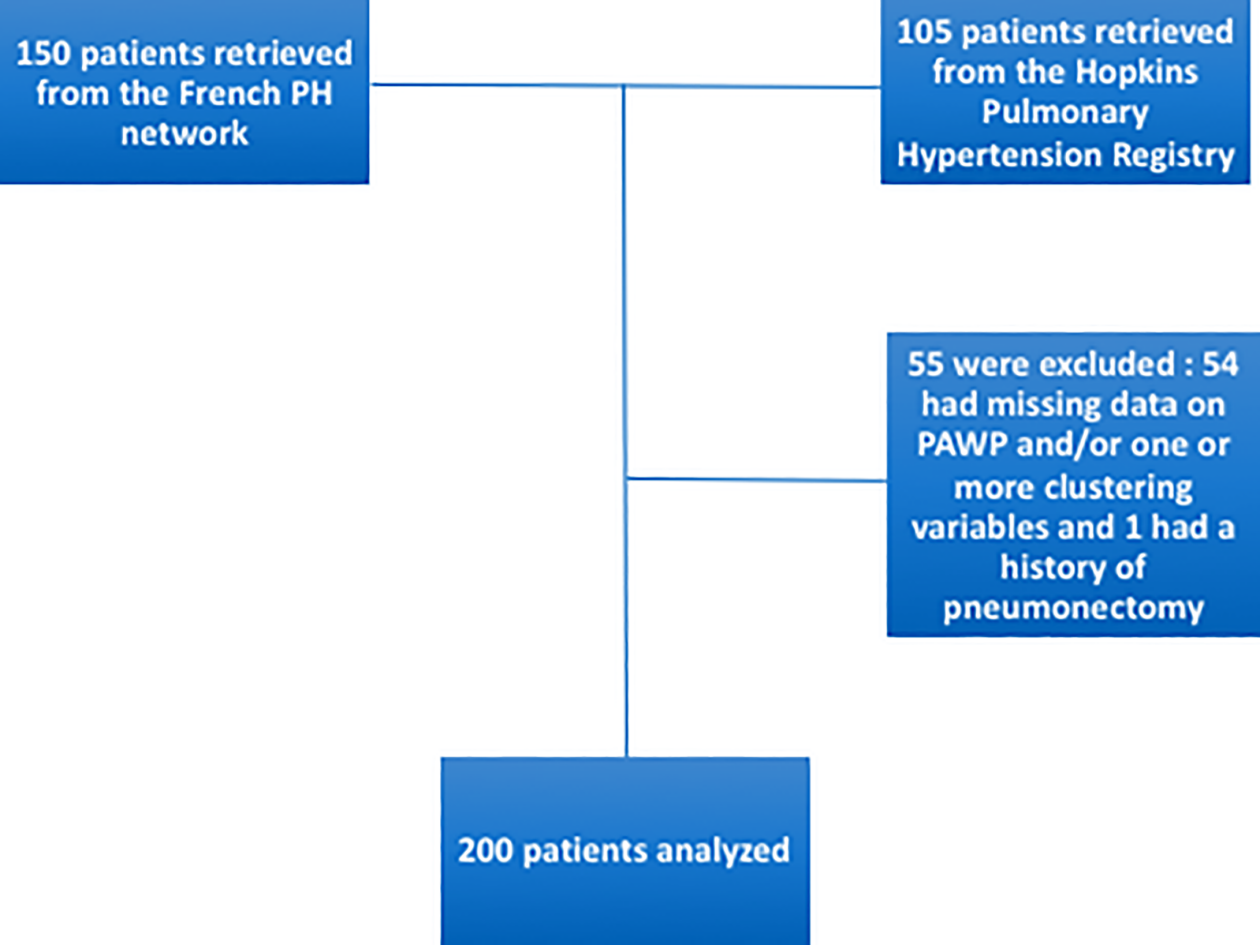
Flowchart of the study.

Patients were included in the present study if they fulfilled the following criteria: (i) age ≥18 years; (ii) ACR/EULAR 2013 criteria for SSc [15]; (iii) no evidence of chronic thrombo-embolic PH; (iv) a calculable follow-up; (v) pre-capillary PH demonstrated by right-heart catheterization with a mean PAP ≥ 25 mmHg and a pulmonary capillary wedge pressure ≤15 mmHg; and (vi) baseline high-resolution computed tomography of the chest and pulmonary functional tests. Chronic thromboembolic PH was ruled out on the basis of a ventilation/perfusion lung scan and computed tomographic pulmonary angiography, when appropriate. The flow-chart of the study is depicted in Fig 1.

### Clinical and radiological variables

The following data were collected in both prospective databases: age at first right-heart catheterization, gender, subtype of SSc[16], New York Heart Association functional class, and six-minute walk distance (6MWD). For pulmonary function tests, the following data were collected: forced vital capacity (FVC), total lung capacity (TLC), diffusing capacity for carbon monoxide (DLCO), forced expiratory volume in one second (FEV1), PaO_2_, PaCO_2_, and SaO_2_ at the end of the 6MWD test. For right-heart catheterization data, we collected mean PAP, pulmonary artery wedge pressure, right atrial pressure, cardiac index, total pulmonary resistance, and pulmonary vascular resistance (PVR).

Each patient had an available high-resolution computed tomography of the chest at the time of PH diagnosis. A diagnosis of ILD was defined by the presence of one or more of the following features: isolated ground-glass opacities, honeycombing with concurrent areas of ground-glass attenuation, and traction bronchiectasis and/or bronchiolectasis. For each patient with ILD on high resolution computed tomography of the chest, the extent of ILD was graded as limited or extensive according to the staging system of Goh et al. [17], by experts (VC and DL for the French cases and PMH, SCM and MS for the US cases). The staging system was established blindly from the clinical data. In cases of discordance between reviewers, the staging was established by consensus.

### Statistical analysis

To delineate homogeneous clusters, clustering based on the K-means method was performed. Clustering variables were chosen on their a priori clinical relevance. As the presence and severity of ILD as well as the severity of PH appeared as the most critical parameters associated with the heterogeneity of PH in SSc, we chose FVC, DLCO, PVR and presence/extent of ILD as clustering variables after standardization. Details of statistical analysis are available in the S1 appendix.

## Results

### Baseline clinical, functional and hemodynamic characteristics

**Fig 2:**
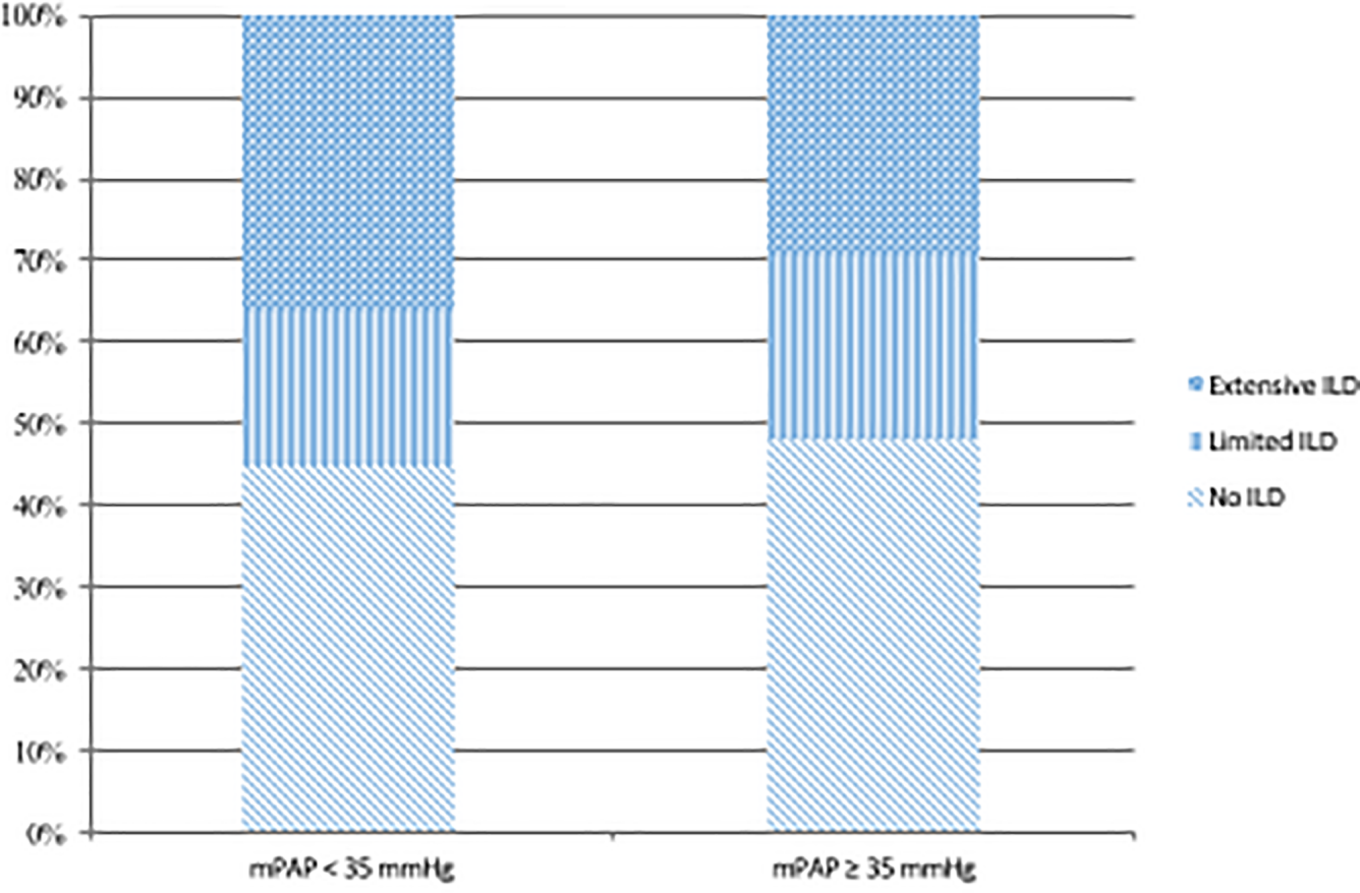
Distribution of patients with no, limited and extensive interstitial lung disease according to mean pulmonary artery pressure.

Two hundred incident SSc patients (124 in the French cohort and 76 in the US cohort) were included in this study (47 males, 23.5%). Mean age was 61.2±11.9 years. The patients’ characteristics are summarized in Table 1. ILD was absent in 94 (47.0%), limited in 42 (21.0%) and extensive in 64 (32.0%) patients. Mean PVR was 8.0±4.6 Wood units. The distribution of the presence and extent of ILD according to the mean PAP is represented in Fig 2.

**Table 1:** Baseline characteristics.

	N	mean±SD or N (%)
Age, years	200	61.2±11.9
Males	200	47 (23.5)
Diffuse systemic sclerosis	198	52 (26.3)
Limited systemic sclerosis	198	146 (73.7)
Anticentromere Ab	128	46 (35.9)
Antitopoisomerase Ab	133	24 (18.1)
NYHA I/II	184	44 (23.9)
NYHA III-IV	184	140 (76.1)
DLCO, % of predicted	200	47.1±18.5
FVC, % of predicted	200	79.2±22.9
FVC/DLCO	200	1.9±1.1
TLC, % of predicted	187	79.2±20.4
FEV1, % of predicted	196	77.5±22.1
PaO_2_, mmHg	96	68.5±16.2
PaCO_2_, mmHg	91	33.8±5.3
PaO_2_+PaCO_2_, mmHg	91	102.2±16.6
6MWD, m	169	286±108
SaO_2_ end of 6MWD, %	110	86.3±7.7
mPAP, mmHg	200	40.4±10.6
PAWP, mmHg	200	8.7±3.4
Cardiac output, L/min	200	4.5±1.4
Cardiac index, L/min/m^2^	200	2.6±0.8
PVR, Wood Units	200	8.0±4.6
RAP, mmHg	117	7.4±4.8
No interstitial lung disease	200	94 (47.0)
Limited ILD	200	42 (21.0)
Extensive ILD	200	64 (32.0)
Limited ILD and mPAP between 25 and 34 mmHg	200	13 (6.5)
Limited ILD and mPAP ≥35 mmHg	200	29 (14.5)
Extensive ILD and mPAP between 25 and 34 mmHg	200	26 (13.0)
Extensive ILD and mPAP ≥35 mmHg	200	38 (19.0)

NYHA: New York Heart Association functional class, DLCO: diffusing capacity for carbon monoxide, FVC: forced vital capacity, TLC: total lung capacity, FEV1: forced expiratory volume in one second, 6MWD: six-minute walk distance, mPAP: mean pulmonary arterial pressure, PAWP: pulmonary artery wedge pressure, PVR: pulmonary vascular resistances, RAP: right atrial pressure, ILD: insterstitial lung disease

### Cluster analysis

Using the Caliński criteria [18], we identified five homogeneous clusters: C1, C2, C3, C4, and C5 (S1 Table and S1 Fig). Clusters C1 and C5 were very similar and were merged in a revised cluster C1 for the final analysis, which therefore included only four clusters (Table 2).

**Table 2:** Baseline characteristics of the four clusters of systemic sclerosis patients with pre-capillary pulmonary hypertension.

		C1 N = 94	C2 N = 61	C3 N = 16	C4 N = 29
Age, years	mean±SD	63.1±11.3	57.2±11.4	60.9±11.7	63.4±13.3
Males	N (%)	23 (24.5%)	20 (32.8%)	2 (12.5%)	2 (6.9%)
Diffuse SSc	N (%)	15 (16.0%)	27 (45.8%)	3 (18.8%)	7 (24.1%)
Anticentromere Ab	N (%)	33 (50.0%)	4 (11.8%)	4 (50.0%)	5 (25.0%)
Antitopoisomerase Ab	N (%)	3 (4.6%)	16 (41.0%)	1 (12.5%)	4 (20.0%)
NYHA III-IV	N (%)	64 (72.7%)	44 (78.6%)	12 (92.3%)	20 (74.1%)
DLCO, % of predicted	mean±SD	45.3±12.6	39.0±15.8	36.9±11.8	75.5±15.8
FVC, % of predicted	mean±SD	90.5±21.9	60.7±16.0	86.4±14.8	77.5±15.6
FVC/DLCO	mean±SD	2.1±0.7	1.8±1.0	2.8±2.2	1.1±0.2
TLC, % of predicted	mean±SD	89.8±17.8	60.3±13.4	82.5±11.4	84.0±16.1
FEV1, % of predicted	mean±SD	86.2±23.0	61.3±15.7	81.9±14.1	80.0±15.8
PaO_2_, mmHg	mean±SD	70.7±18.3	64.0±9.5	56.9±15.7	73.5±16.1
PaCO_2_, mmHg	mean±SD	31.8±4.2	37.2±5.2	29.6±2.4	36.1±5.8
PaO_2_+PaCO_2_, mmHg	mean±SD	102.1±18.5	101.4±10.6	86.4±15.8	110.7±16.2
6MWD, m	mean±SD	299±112	276±100	237±120	290±104
SaO_2_ end of 6MWD, %	mean±SD	86.7±6.4	82.1±9.6	83.1±5.9	91.7±5.0
mPAP, mmHg	mean±SD	40.2±9.9	37.4±8.6	55.3±9.4	39.1±10.9
PAWP, mmHg	mean±SD	8.3±3.7	9.0±3.4	8.3±2.8	9.8±2.8
Cardiac output, L/min	mean±SD	4.5±1.4	4.8±1.3	2.6±0.5	5.0±1.2
Cardiac index, L/min/m^2^	mean±SD	2.6±0.7	2.8±0.8	1.6±0.3	3.0±0.7
PVR, Wood Units	mean±SD	7.8±3.2	6.4±3.2	18.7±4.9	6.1±2.4
RAP, mmHg	mean±SD	7.7±4.9	6.1±4.0	11.0±4.4	8.5±5.9
No ILD	N (%)	67 (71.3%)	0	5 (31.3%)	22 (75.9%)
Limited ILD	N (%)	25 (26.6%)	0	10 (62.5%)	7 (24.1%)
Extensive ILD	N (%)	2 (2.1%)	61 (100.0%)	1 (6.3%)	0
Limited ILD and mPAP between 25 and 34 mmHg		9 (9.6%)	0	0	4 (13.8%)
Limited ILD and mPAP ≥35 mmHg		16 (17.0%)	0	10 (62.5%)	3 (10.3%)
Extensive ILD and mPAP between 25 and 34 mmHg		0	26 (42.6%)	0	0
Extensive ILD and mPAP ≥35 mmHg		2 (2.1%)	35 (57.4%)	1 (6.3%)	0

NYHA: New York Heart Association functional class, DLCO: diffusing capacity for carbon monoxide, FVC: forced vital capacity, TLC: total lung capacity, FEV1: forced expiratory volume in one second, 6MWD: six-minute walk distance, mPAP: mean pulmonary arterial pressure, PAWP: pulmonary artery wedge pressure, PVR: pulmonary vascular resistances, RAP: right atrial pressure, ILD: insterstitial lung disease

Cluster C1 (n = 94) was mainly characterized by pre-capillary PH with the majority of patients having limited or no ILD (mild to moderate risk PAH). Mean FVC was normal while DLCO was low (45.3±12.6%). In this cluster, we found the lowest proportion of antitopoisomerase 1 antibodies and diffuse SSc subset.

Cluster C2 (n = 61) was mainly characterized by the presence of an extensive ILD in all patients and mild to moderate risk pre-capillary PH (PVR: 6.4±3.2 Wood Units). This cluster was also characterized by the lowest FVC (60.7±16.0% predicted), a low DLCO (39.0±15.8% predicted), the highest percentage of antitopoisomerase 1 antibodies and diffuse SSc subset. This was also the youngest cluster.

Cluster C3 (n = 16) was characterized by severe pre-capillary PH with a low cardiac index and a majority of patients with limited or no ILD (93.8%) (severe PAH). Mean FVC was normal with a low DLCO (36.9±11.8% predicted). This cluster was also characterized by a high percentage of anticentromere antibodies, with a low percentage of the diffuse SSc subset, and a low percentage of males.

**Fig 3:**
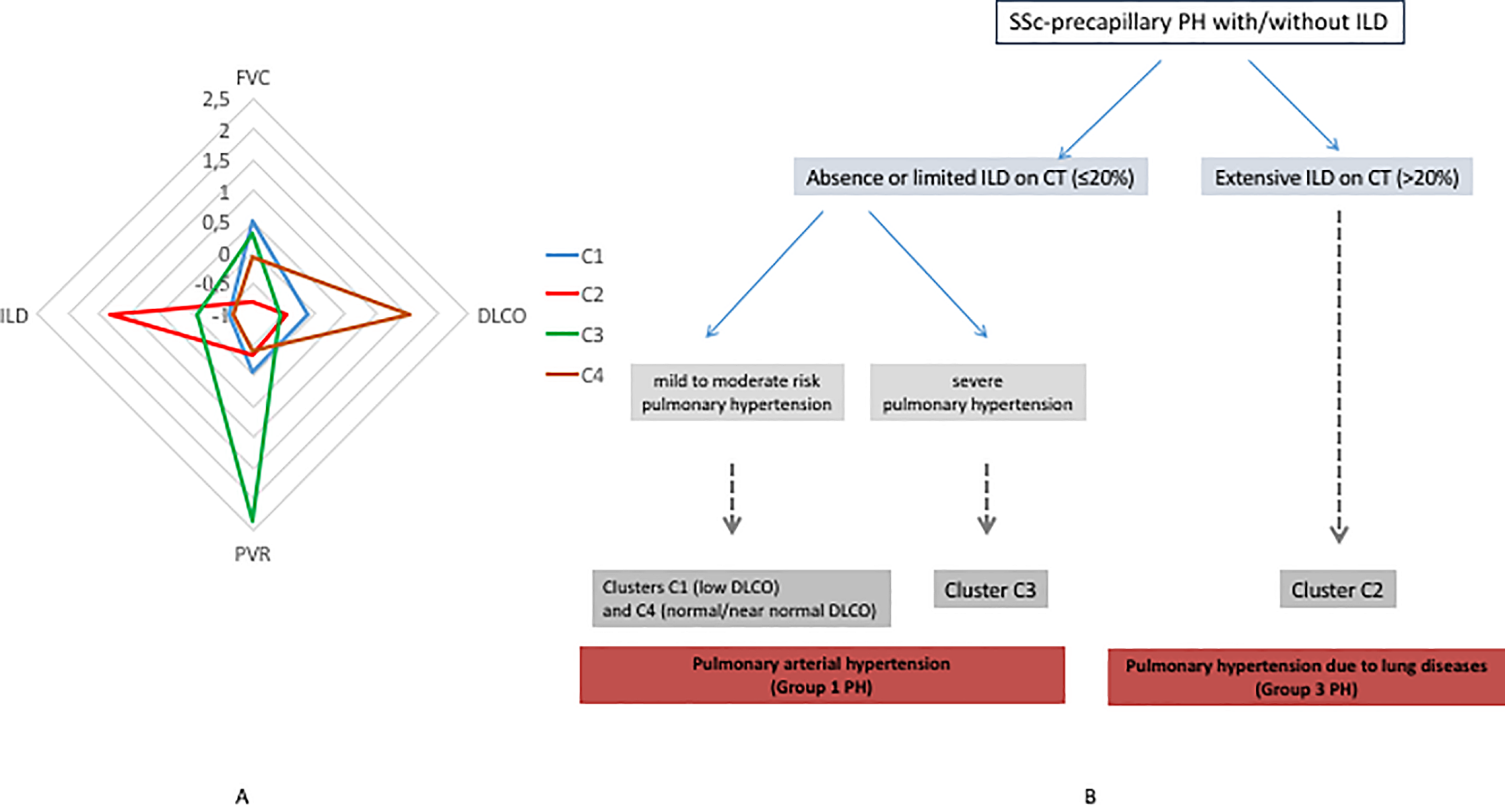
A. Radar plot of the four clusters according to clinical characteristics, presence and severity of interstitial lung disease and severity of hemodynamics. B. Algorithm of classification in the four clusters C1, C2, C3 and C4.

Cluster C4 (n = 29) was characterized by less pronounced pre-capillary PH with all patients having either limited or no ILD (mild to moderate risk PAH). Mean DLCO was normal or near-normal (75.5±15.8%). The percentage of diffuse SSc and antitopoisomerase 1 antibodies was relatively high, contrasting with the lowest percentage of males (6.9%). Both C1 and C4 included older patients. To best visualize the differences between clusters, we constructed a radar plot using means of the clustering variables (Fig 3A) and an algorithm (Fig 3B)

### Survival

**Fig 4:**
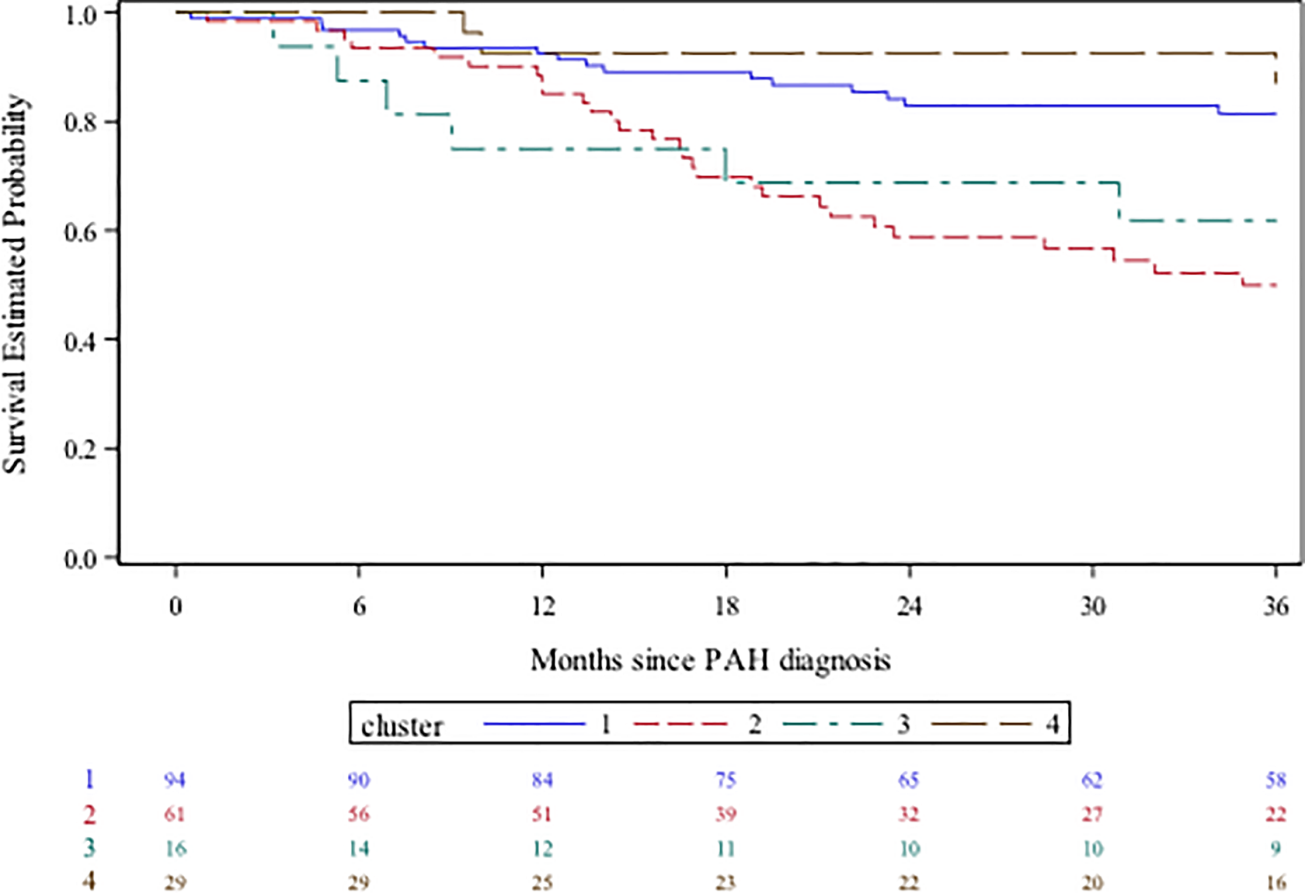
Survival of the four clusters C1, C2, C3 and C4. The difference of survival between the 4 clusters was significant (p = 0.0002).

The overall survival was 73.6% at three years (S2 Fig) with marked differences between the 4 clusters: 81.5% [95% confidence interval 71.4–88.2] for C1, 49.9% [35.9–62.4] for C2, 61.9% [33.9–80.8] for C3, and 87.1% [64.5–97.8] for C4 (p = 0.0002 between the 4 clusters; Fig 4). After adjusting for age and sex, when compared to C1 as a reference, C2 had a significantly worse prognosis (C2: adjusted hazard ratio for death: 3.14 [95% CI 1.66–5.94], p = 0.0004) and there was a trend towards worse outcomes for C3 (2.53 [95% CI 0.99–6.49], p = 0.052). There was no difference with C4 (0.65 [95% CI 0.19–2.27], p = 0.507). Statistical power was 99.4% for comparison between C1 and C2, 24% for comparison of C1 to C3 and 14.5% for comparison of C1 to C4.

## Discussion

SSc is a heterogeneous disease with a range of skin extension, organ involvement and autoantibody status [8]. Pre-capillary PH in SSc is similarly heterogeneous, as patients may present with a spectrum of clinical phenotypes, ranging from pure PAH without parenchymal lung involvement to PH due to extensive ILD [9, 19, 20]. In daily practice, many SSc patients are difficult to classify and lie somewhere between these two extreme presentations. In this complex clinical context combining the heterogeneity of SSc and PH, it is of paramount importance to best classify pre-capillary PH in order to try to predict future risk and to identify the optimal management strategy. Our study aimed to address these issues by using cluster analysis to identify homogeneous groups amongst SSc patients with pre-capillary PH and compare their survival.

Our analysis identified four simple homogeneous groups, which differed in terms of clinical presentation, hemodynamic severity, presence and extent of ILD, as well as survival. These results allow us to deconstruct the wide heterogeneity of pre-capillary PH in SSc into clinically relevant and simple groups. The main characteristics of the clusters can be summarized as follows: cluster C1 corresponds to “mild to moderate risk PAH without extensive ILD and with a low DLCO”. Cluster C2 is predominantly characterized by “pre-capillary PH due to extensive ILD and with a low DLCO”; cluster C3 is characterized by “severe PAH without extensive ILD and with a low DLCO”; and cluster C4 can be described as “mild to moderate risk PAH without extensive ILD and with a normal DLCO”. The two main consequences of these 4 clusters is that 1. The presence of an extensive ILD, whatever the hemodynamics, is associated with a very severe outcome 2. The presence of a limited ILD (often seen as a potent cofounder the classification of PH in tne context of SSc) has to be considered in the same group of patients with no ILD, where the severity of hemodynamics drives the prognosis.

One cluster (C2) was mainly characterized by the presence of extensive ILD in all patients while the other clusters had either no or a limited ILD. While this result could appear as predictable, it must be highlighted that the cluster analysis did not identify two different groups of patients with extensive ILD and, for example, different hemodynamics or DLCO. This suggests that the presence of an extensive ILD is an important discriminative characteristic *per se* while the presence of a limited ILD is less relevant to categorization. Moreover, this result also suggests that differentiating patients with pre-capillary PH and extensive ILD according to the level of mean PAP or the severity of other hemodynamic variables may not be useful. Thus, cluster C2 corresponds to group 3 of the PH classification [2, 3] while patients with limited ILD are closer to patients without ILD and, therefore, have group 1 PAH. Patients in C2 had the worst survival at three years (49.9% [35.9–62.4]), in line with a recent meta-analysis [6].

Cluster C3 patients were characterized by severe PAH without extensive ILD. Compared to the other clusters, PVR was the highest, and cardiac index the lowest. Consistent with the known poor prognostic features of low cardiac index and low DLCO, this cluster carried a poor prognosis with a 3-year survival of 61.9% [95% confidence interval 33.9–80.8]. These patients could correspond to the most severe form of group 1 but could also gather patients with pulmonary veno-occlusive disease-like disease (group 1’) explaining both their severity and poor prognosis.

Clusters C1 and C4 were rather close, gathering patients with mild to moderate risk PAH with the majority of patients having no or limited ILD. Of note, these two clusters aggregated 123/200 patients and correspond to the most common presentation of pre-capillary PH in SSc. However, there were some differences between C1 and C4. One difference was that C4 patients had a normal DLCO while C1 patients had a low DLCO. It remains important for clinicians to recognize that within these non-severe group 1 PAH, the presence of a low DLCO may identify patients at a higher risk of progression and mortality. Three-year survival of these two clusters ranged from 81% to 87%, which is much better than the survival of other clusters.

An unanswered issue is whether C3, C1 and C4 are distinct clusters or a same cluster diagnosed at a different stage of their disease. While we cannot draw any firm conclusion, C3 includes younger patients with a very severe PH and seems to be a distinct and aggressive phenotype.

Despite the potential clinical utility of the phenotypic clusters identified in this study, certain limitations must be acknowledged. First, although patients from both the French and US Registries were prospectively enrolled, the data collection and analysis were retrospective with some missing data encountered and, therefore, is potentially prone to bias. Second, data on initial treatment strategy and causes of death for patients with were not available. As data on treatment are lacking, we cannot rule out the hypothesis that the clusters with the worst survival could be characterized by a poorer response to specific PH treatment. Third, one could argue that the observed results could have been easily anticipated with obvious clusters. However, our results provide important clues to know how to classify patients with extensive ILD whatever the hemodynamics as well as how to deal with patients with limited ILD, two important issues, which are often debated in this complication of SSc. Moreover, the use of an incident cohort, validation across two patient cohorts and the face validity of our findings are all notable, but a validation in a second independent dataset of patients would strengthen this study. Fourth, we did not include post-capillary PH, which could be a weakness for generalizability of our results.

In conclusion, our study allowed us to decipher the heterogeneity of PH in patients with SSc. Four homogeneous groups were identified. Two carried a dismal prognosis, one characterized by the presence of extensive ILD and another by severely impaired hemodynamics. It is probable that these two clusters would benefit from future efforts to improve their management, including early referral to lung transplantation centers. The two other clusters were characterized by either the absence of ILD or the presence of limited ILD, with mild to moderate risk PAH and a relatively favorable overall prognosis. Our attempt to clarify the heterogeneity of this disease should help clinicians to anticipate the prognosis of patients and guide individual management.

## Supporting information

S1 Appendix Statistical analysis. (DOCX)

S1 Fig Scatter plot of patients against canonical dimensions derived from a discriminant analysis. Patients are represented by the number of the cluster to which they belong.
(PDF)

S2 Fig Overall survival of 200 systemic sclerosis patients with pre-capillary pulmonary hypertension. (PPTX)

S1 Table Baseline characteristics of the five clusters of systemic sclerosis patients with pre-capillary pulmonary hypertension. (DOCX)
