# Supplementary material for: Clinical phenotypes and survival of pre-capillary pulmonary hypertension in systemic sclerosis
Source: PLoS One. 2018 May 15;13(5):e0197112. doi: 10.1371/journal.pone.0197112 (PMC5953495; doi:10.1371/journal.pone.0197112)
Supplement: S1 Appendix — (DOCX) [file pone.0197112.s001.docx]

e-Appendix 1.

Statistical analysis.

To delineate homogeneous clusters, clustering based on the K-means method was performed using four clustering variables after standardization: forced vital capacity, diffusing capacity for carbon monoxide, pulmonary vascular resistance and presence/extent of interstitial lung disease. Clustering variables were chosen on their *a priori* clinical relevance. The number of clusters was chosen on the values of R square, Cluster Cubic Criterion and pseudo-F statistic ^1^. The K-means method uses the k first observations as seeds for the classification and therefore results may depend on the way observations are ordered. We performed 500 iterations of random sorting of the dataset and cluster analysis and we described the number of times some observations were grouped together. As expected, most observations were never or almost always grouped (data not shown). Additionally, we used the Ward method for clustering observations. This method does not depend on the way observations are sorted. Since both methods gave roughly the same results, we only present the results of the k-means method. Survival curves were plotted by the Kaplan-Meier method. Crude and adjusted (for age at diagnosis and gender) Cox models were performed. Assumption of proportional hazards was checked by graphical methods (plot of ‘log-negative-log’ of the Kaplan Meier estimator of the survival function, plot of Schoenfeld residuals) and by introduction in the model of interaction of each covariate with time. Since interaction with time was far from statistical significance and Akaike Criterion was not improved, interaction terms were removed from the final model.

The statistical analysis was conducted with the SAS 9.4 software (SAS Institute, Cary, NC, US).

REFERENCES

1. Caliński T, Harabasz J. A Dendrite Method for Cluster Analysis. Communications in Statistics 1974; 3:1–27.
