# Supplementary figures and images for: Clinical phenotypes and survival of pre-capillary pulmonary hypertension in systemic sclerosis
Source: PLoS One. 2018 May 15;13(5):e0197112. doi: 10.1371/journal.pone.0197112 (PMC5953495; doi:10.1371/journal.pone.0197112)

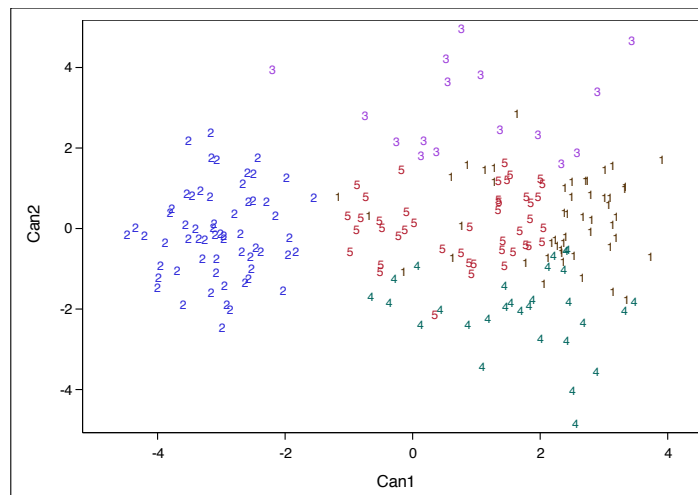

Supplemental Figure 1

Supplement: S1 Fig — Patients are represented by the number of the cluster to which they belong. (PDF) [file pone.0197112.s002.pdf]

## Slide 1
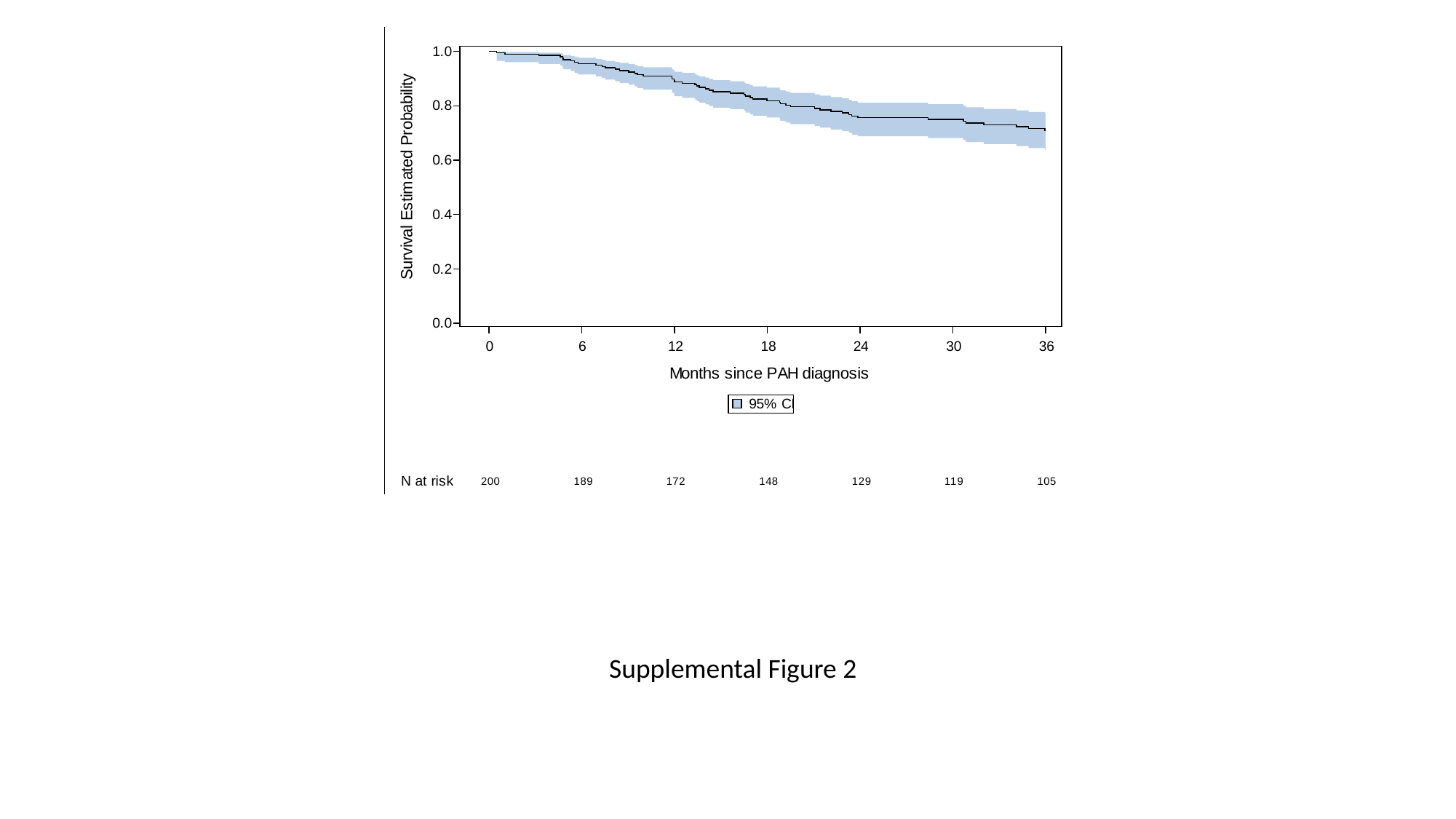

Supplemental Figure 2

Supplement: S2 Fig — (PPTX) [file pone.0197112.s003.pptx]
