# Supplementary material for: Clinical phenotypes and survival of pre-capillary pulmonary hypertension in systemic sclerosis
Source: PLoS One. 2018 May 15;13(5):e0197112. doi: 10.1371/journal.pone.0197112 (PMC5953495; doi:10.1371/journal.pone.0197112)
Supplement: S1 Table — (DOCX) [file pone.0197112.s004.docx]

e-Table 1

|  |  | C1  N=50 | C2  N=61 | C3  N=16 | C4  N=29 | C5  N=44 |
| --- | --- | --- | --- | --- | --- | --- |
| age, yrs | m±SD | 65.0±11.6 | 57.2±11.4 | 60.9±11.7 | 63.4±13.3 | 61.0±10.7 |
| males | N (%) | 11 (22.0%) | 20 (32.8%) | 2 (12.5%) | 2 (6.9%) | 12 (27.3% |
| diffuse SSc | N (%) | 8 (16.0%) | 27 (45.8%) | 3 (18.8%) | 7 (24.1%) | 7 (15.9%) |
| anticentromere Ab | N (%) | 21 (56.8%) | 4 (11.8%) | 4 (50.0%) | 5 (25.0%) | 12 (41.4%) |
| antitopoisomerase Ab | N (%) | 2 (5.4%) | 16 (41.0%) | 1 (12.5%) | 4 (20.0%) | 1 (3.5%) |
| NYHA III | N (%) | 31 (66.0%) | 33 (58.9%) | 6 (46.2%) | 20 (74.1%) | 25 (61.0%) |
| NYHA IV | N (%) | 4 (8.5%) | 11 (19.6%) | 6 (46.2%) | 0 | 4 (9.8%) |
| NYHA III-IV | N (%) | 35 (74.5%) | 44 (78.6%) | 12 (92.3%) | 20 (74.1%) | 29 (70.7%) |
| DLCO, % of predicted | m±SD | 52.4±10.5 | 39.0±15.8 | 36.9±11.8 | 75.5±15.8 | 37.2±9.5 |
| FVC, % of predicted | m±SD | 107.0±13.1 | 60.7±16.0 | 86.4±14.8 | 77.5±15.6 | 71.6±12.6 |
| FVC/DLCO |  | 2.1±0.6 | 1.8±1.0 | 2.8±2.2 | 1.1±0.2 | 2.1±0.9 |
| TLC, % of predicted | m±SD | 97.7±13.7 | 60.3±13.4 | 82.5±11.4 | 84.0±16.1 | 81.0±17.9 |
| FEV1, % of predicted | m±SD | 102.3±14.5 | 61.3±15.7 | 81.9±14.1 | 80.0±15.8 | 67.4±15.6 |
| PaO2, mmHg | m±SD | 71.3±16.1 | 64.0±9.5 | 56.9±15.7 | 73.5±16.1 | 69.8±21.4 |
| PaCO2, mmHg | m±SD | 32.3±4.3 | 37.2±5.2 | 29.6±2.4 | 36.1±5.8 | 31.1±4.1 |
| PaO2+PaCO2, mmHg | m±SD | 103.8±15.7 | 101.4±10.6 | 86.4±15.8 | 110.7±16.2 | 99.8±22.0 |
| 6 MWD, m | m±SD | 292±107 | 276±100 | 237±120 | 290±104 | 307±118 |
| SaO2 end of 6MWD, % | m±SD | 87.9±5.9 | 82.1±9.6 | 83.1±5.9 | 91.7±5.0 | 85.1±6.7 |
| mPAP, mmHg | m±SD | 40.2±10.4 | 37.4±8.6 | 55.3±9.4 | 39.1±10.9 | 40.1±9.3 |
| PAWP, mmHg | m±SD | 8.4±3.6 | 9.0±3.4 | 8.3±2.8 | 9.8±2.8 | 8.1±3.7 |
| cardiac index, L/min/m^2^ | m±SD | 2.7±0.8 | 2.8±0.8 | 1.6±0.3 | 3.0±0.7 | 2.5±0.5 |
| cardiac output, L/min | m±SD | 4.7±1.6 | 4.8±1.3 | 2.6±0.5 | 5.0±1.2 | 4.3±1.0 |
| PVR, Woods U | m±SD | 7.6±3.5 | 6.4±3.2 | 18.7±4.9 | 6.1±2.4 | 7.9±3.0 |
| RAP, mmHg | m±SD | 7.7±5.0 | 6.1±4.0 | 11.0±4.4 | 8.5±5.9 | 7.7±4.9 |
| no ILD on HRCT scan | N (%) | 38 (76.0%) | 0 | 5 (31.3%) | 22 (75.9%) | 29 (65.9%) |
| limited ILD | N (%) | 10 (20.0%) | 0 | 10 (62.5%) | 7 (24.1%) | 15 (34.1%) |
| extensive ILD | N (%) | 2 (4.0%) | 61 (100.0%) | 1 (6.3%) | 0 | 0 |
| Limited ILD and mPAP between 25 and 34 mmHg | N (%) | 4 (8.0%) | 0 | 0 | 4 (13.8%) | 5 (11.4%) |
| Limited ILD and mPAP ≥35 mmHg | N (%) | 6 (12.0%) | 0 | 10 (62.5%) | 3 (10.3%) | 10 (22.7%) |
| Extensive ILD and mPAP between 25 and 34 mmHg | N (%) | 0 | 26 (42.6%) | 0 | 0 | 0 |
| Extensive ILD and mPAP ≥35 mmHg | N (%) | 2 (4.0%) | 35 (57.4%) | 1 (6.3%) | 0 | 0 |

NYHA : New York Heart Association functional class, DLCO : diffusing capacity for carbon monoxide, FVC : forced vital capacity, TLC : total lung capacity, FEV1 : forced expiratory volume in one second, 6MWD : six-minute walk distance, mPAP : mean pulmonary arterial pressure, PAWP : pulmonary artery wedge pressure, PVR : pulmonary vascular resistances, RAP : right atrial pressure, ILD : insterstitial lung disease
